# Bovine Milk Extracellular Vesicles Are Osteoprotective by Increasing Osteocyte Numbers and Targeting RANKL/OPG System in Experimental Models of Bone Loss

**DOI:** 10.3389/fbioe.2020.00891

**Published:** 2020-07-31

**Authors:** Marina C. Oliveira, Bartijn C. H. Pieters, Polianna B. Guimarães, Letícia F. Duffles, Joyce E. Heredia, Ana L. M. Silveira, Amanda C. C. Oliveira, Mauro M. Teixeira, Adaliene V. M. Ferreira, Tarcilia A. Silva, Fons A. J. van de Loo, Soraia Macari

**Affiliations:** ^1^Laboratory of Immunometabolism, Department of Nutrition, Nursing School, Universidade Federal de Minas Gerais, Belo Horizonte, Brazil; ^2^Experimental Rheumatology, Radboud University Medical Center, Nijmegen, Netherlands; ^3^Laboratory of Immunopharmacology, Department of Biochemistry and Immunology, Institute of Biological Sciences, Universidade Federal de Minas Gerais, Belo Horizonte, Brazil; ^4^Department of Oral Surgery and Pathology, Faculty of Dentistry, Universidade Federal de Minas Gerais, Belo Horizonte, Brazil; ^5^Department of Restorative Dentistry, Faculty of Dentistry, Universidade Federal de Minas Gerais, Belo Horizonte, Brazil

**Keywords:** extracellular vesicles, milk, bone loss, osteoporosis, obesity, osteocyte

## Abstract

Studying effects of milk components on bone may have a clinical impact as milk is highly associated with bone maintenance, and clinical studies provided controversial associations with dairy consumption. We aimed to evaluate the impact of milk extracellular vesicles (mEVs) on the dynamics of bone loss in mice. MEVs are nanoparticles containing proteins, mRNA and microRNA, and were supplemented into the drinking water of mice, either receiving diet-induced obesity or ovariectomy (OVX). Mice receiving mEVs were protected from the bone loss caused by diet-induced obesity. In a more severe model of bone loss, OVX, higher osteoclast numbers in the femur were found, which were lowered by mEV treatment. Additionally, the osteoclastogenic potential of bone marrow-derived precursor cells was lowered in mEV-treated mice. The reduced stiffness in the femur of OVX mice was consequently reversed by mEV treatment, accompanied by improvement in the bone microarchitecture. In general, the RANKL/OPG ratio increased systemically and locally in both models and was rescued by mEV treatment. The number of osteocytes, as primary regulators of the RANKL/OPG system, raised in the femur of the OVX mEVs-treated group compared to OVX non-treated mice. Also, the osteocyte cell line treated with mEVs demonstrated a lowered RANKL/OPG ratio. Thus, mEVs showed systemic and local osteoprotective properties in two mouse models of bone loss reflected in reduced osteoclast presence. Data reveal mEV potential in bone modulation, acting via osteocyte enhancement and RANKL/OPG regulation. We suggest that mEVs could be a therapeutic candidate for the treatment of bone loss.

## Introduction

Osteoporosis is a multifactorial disease characterized by low bone mass and structural deterioration of bone tissue, leading to fragility and consequently increased risk of fractures (Rolland et al., 2008). Although osteoporosis occurs in both sexes, it is a prevalent disease in women and usually related to the postmenopausal period (Tarity et al., 2013). Typically, estrogen exerts its function through specific receptors, promoting apoptosis of osteoclasts (Cauley, 2015) and increasing the differentiation of osteoblasts (Hughes et al., 1996). Moreover, bone loss can also be associated with other diseases, such as obesity (Cao, 2011). Obesity is characterized by an expansion of adipose tissue and chronic inflammation, usually developed from nutrient overloads (Greenberg and Obin, 2006). The unfavorable nutritional environment with the reduction in the intake of calcium and vitamins compromises the maintenance of bone health (Keller et al., 2009). Also, pro-inflammatory cytokines from obesity-induced chronic inflammation disrupt the bone homeostasis by activating bone-resorbing cells and inhibiting osteoblasts (Ornstrup et al., 2015). Therefore, these two scenarios, osteoporosis and obesity, are quite common to be observed in the population and closely related to bone loss development and, consequently, reduced bone quality.

The OPG/RANKL/RANK system regulates cell function through the control of osteoclastogenesis and bone remodeling. RANKL binds to its receptor RANK, stimulating osteoclast activation and survival (Boyle et al., 2003). On the other side, OPG is derived primarily from cells of the osteoblast lineage and is described as an antagonist of RANKL by competing with RANK for the binding of RANKL (Lacey et al., 1998, 2000). Osteocytes, the end stage of osteoblast differentiation, are a rich source of OPG and RANKL, and by their differential production, these cells can regulate osteoclast-mediated bone resorption (Goldring, 2015). Modulation of this system is a crucial mechanism for maintaining an adequate balance between bone formation and resorption. Despite knowing the leading cause of bone loss, studies regarding the effect of food components in this disease are still necessary.

Milk is a nutritious fluid recommended for improving bone health. However, studies have shown a controversial relation regarding the consumption of milk or its components and the maintenance of bone tissue (Feskanich et al., 2003; Kalkwarf et al., 2003; Uenishi et al., 2007; Bonjour et al., 2008; Michaëlsson et al., 2014). Studies demonstrate positive aspects of milk consumption by showing its association with increased bone density in women and children (Kalkwarf et al., 2003; Uenishi et al., 2007). In postmenopausal women, a reduction in bone turnover was observed after 6 months of supplementation with milk (Bonjour et al., 2008). However, the consumption of milk more than three times a day has been associated with the risk of fractures in men and women, with the presence of galactose in its composition being referred (Michaëlsson et al., 2014). Another study showed that only vitamin D consumption was associated with a lower risk of osteoporosis fractures, the same not being observed for milk and calcium in postmenopausal women (Feskanich et al., 2003). Recent studies related to milk components highlight the extracellular vesicles (EVs) (Aqil et al., 2019; Benmoussa et al., 2019, 2020; Zeng et al., 2019). EVs are membranous vesicles released by the cells into the extracellular medium, with a size smaller than 300 nm. Their origin may be endosomal, cytoplasm or plasma-membrane termed exosomes or microvesicles, respectively. Studies have shown that EVs contain proteins, mRNA and microRNA (miRNA) (Izumi et al., 2012; Raposo and Stoorvogel, 2013; Hartjes et al., 2019). Adequate identification of the effect of milk components may have a significant clinical impact since EVs are still present in commercial cow milk even after the pasteurization process (Raposo and Stoorvogel, 2013). Currently, strategies have been used to study the role of EVs, especially *in vitro*, demonstrating their ability to interact with other cells and modulate their functionality (Qin et al., 2016; Ragni et al., 2019). However, studies *in vivo* are still lacking, and this needs to be explored further.

In the last years, only a few studies demonstrated the implication of extracellular vesicles obtained from commercial bovine milk (mEVs) on bone cells (Oliveira et al., 2016, 2017). It was observed that healthy female mice after oral consumption of mEVs increased the number of osteocytes and the presence of bone considered immature (Oliveira et al., 2016). Also, there was an increase in the presence of osteoclasts but no change in RANKL and CTX-I *in vivo*, two markers of resorptive activity in the bone (Oliveira et al., 2016). Complementary to these data, *in vitro* analyses demonstrated an increase in the differentiation of human mesenchymal stem cells into osteoblasts, and mice bone marrow cells into osteoclasts after mEV stimulus, but the latter with a reduction of the bone reabsorbed area (Oliveira et al., 2017). Thus, these data already demonstrated the potential of mEVs to influence cells related to bone remodeling and indicate the potential for a new therapy to prevent bone loss.

Herein, we aimed to evaluate the impact of orally administered mEVs on the dynamics of bone loss using two models: diet-induced obesity and ovariectomized mice. We showed that treatment with mEVs improved the bone microarchitecture in the diet-induced obese mice. In the osteoporotic model, mEVs reduced the presence of osteoclasts and improved bone parameters in the femur, which were correlated with a systemic and local reduction of the RANKL/OPG ratio. In agreement with these findings, we showed that osteocytes treated with mEVs *in vitro* also have a modulated RANKL/OPG expression.

## Materials and Methods

### Diet-Induced Obesity Model and Oral Glucose Tolerance Test

Male BALB/c mice (*n* = 21) 8 weeks of age were provided by the Bioterism Center of the Universidade Federal de Minas Gerais (UFMG). They were maintained in a controlled environment with 12 h:12 h light:dark cycle, and in collective cages with *ad libitum* access to food and water. The “Ethics Committee for Animal Experimentation of the UFMG” (protocol number: 23/2019) approved the experiments.

The mice were randomly divided into two groups and received the following diets: a standard chow diet (4 kcal/g) as the control group (C) (*n* = 7), and a high refined carbohydrate-containing (HC) diet (4.4 kcal/g) to induce obesity (*n* = 14), during 12 weeks. The HC diet was composed of 45% condensed milk, 10% refined sugar (at least 30% – mostly sucrose), and 45% powder of chow diet. HC diet is a normocaloric diet compared to control, which induces mild obesity, considering that mice have higher adiposity without increasing body weight (Oliveira et al., 2013). The content of calcium of these diets is not different (C: 1.8 ± 0.001 vs. HC: 1.9 ± 0.010 g/100 g; *P* < 0.05). Body mass gain was measured once a week, and the food intake was measured twice a week. From week 8 until week 12, mice were treated with mEVs (14.3 × 10^6^ particles/mL) in their drinking water (*n* = 7). The drinking water was completely replaced every 2–3 days.

For the oral glucose tolerance test (OGTT), D-glucose (2 mg/g body weight) was given orally to fasted mice (6 h) at week 11. Glucose levels were monitored from tail blood samples at 0, 15, 30, 60, 90, and 120 min after glucose overload using an Accu-Chek glucometer (Roche Diagnostics, Indianapolis, IN, United States).

After mice were killed, samples of blood were collected for further analyses as well as visceral adipose tissues (epididymal, retroperitoneal, and mesenteric) and subcutaneous (inguinal), then were weighed.

### Osteoporosis Model

Ten-week-old female C57BL/6 mice (*n* = 32) were obtained from the Bioterism Center of Universidade Federal de Minas Gerais (UFMG). All animals were kept in a controlled environment with 12 h:12 h light:dark cycle, and in collective cages. They were also maintained according to the ethical guidelines of our institution (UFMG) (protocol number 2/2017). Animals were divided into the following groups: mice treated with PBS and (i) pseudo-operated (C-SHAM) (*n* = 8) or (ii) ovariectomized (C-OVX) (*n* = 8) and mice receiving mEVs in the drinking water (14.3 × 10^6^ particles/mL) (iii) pseudo-operated (mEVs-SHAM) (*n* = 8) or (iv) ovariectomized (mEVs-OVX) (*n* = 8). The animals received food and water with the respective treatment *ad libitum* for 5 weeks. The drinking water was completely replaced every 2–3 days. Mice were bilaterally ovariectomized under an anesthetic condition with ketamine (80 mg/kg) and xylazine (10 mg/kg) after 7 days of mEV treatment, starting to generate an estrogen−deficiency model of osteoporosis. At the end of the treatment, animals were anesthetized and killed 30 days after ovariectomy summing up 5 weeks of mEV treatment. Samples of blood, uterus, and femur were collected for further analyses.

### Bovine Milk Extracellular Vesicles Isolation and Characterization

Bovine mEVs were isolated from commercial semi-skimmed cow milk and characterized, as previously described (Arntz et al., 2015; Pieters et al., 2015; Oliveira et al., 2016). In short, milk was centrifuged at 70,000 × *g* for 1 h at 4°C to remove fat globules, proteins, and other debris. The transparent part of the supernatant was filtered through a Whatman paper nr.1, followed by nr.50. MEVs were isolated from the filtered supernatant by ultracentrifugation at 110,000 × *g* for 90 min at 4°C without breaks. MEVs, located on top of a firm casein pellet, were washed and taken up in PBS or the appropriate buffer for RNA or protein analysis. Only traces of calcium was detected (48 ppm). MEVs were extensively characterized based on morphology, size, protein and miRNA content and meet the minimal criteria set up by the ISEV for extracellular vesicles (Théry et al., 2018).

Bovine milk is a rich source of extracellular vesicles, and it is known that these molecules are highly stable and resistant to RNAse activity, extreme pH and temperature (Izumi et al., 2012; Pieters et al., 2015), indicating resistance to similar conditions present in the gastrointestinal tract. Using differential ultracentrifugation EVs were isolated, averaging (4.0 ± 0.9)xe9 particles per milliliter of pasteurized milk, with a mean particle diameter of 130 nm, matching the previously described size for exosome-like vesicles (Somiya et al., 2018). Further characterization of the milk EVs confirmed floating density between 1.15 and 1.21 g/ml (sucrose), and the presence of CD81 and HSP70, two classical EV-markers.

### Micro-Computed Tomography Analysis

Femur samples were fixed in 10% neutral buffered formalin for 48 h and scanned using a microCT system (Skyscan 1172 X-Ray microtomography; Skyscan, Aartselaar, Belgium). The calibration was carried out with known density calcium hydroxyapatite phantoms (Skyscan, Aartselaar, Belgium). High-resolution images with an isotropic voxel size of 8.62 were acquired (50 kV, 0.5-mm aluminum filter) and the trabecular bone in the metaphyseal region of femurs with an irregular anatomic region of interest adjacent to the endocortical boundary was delineated. The tissues were analyzed to determine the bone mineral density (BMD), percent bone volume/tissue volume (BV/TV), trabecular separation (Tb.Sp), trabecular thickness (Tb.Th), trabecular thickness distribution (Tb.Th distribution), trabecular number (Tb.N), cortical percentage of bone volume/tissue volume (Ct.BV/TV), and cortical thickness (Ct.Th).

### Mechanical Analysis

Mechanical analysis of the femur was determined as previously described (Yanagihara et al., 2016). Maximum load and stiffness were determined by testing femurs to fracture in a universal testing machine (EMICs, DL 10000, Ribeirão Preto, Brazil) equipped with a load cell of 500 N, and TESC software, version 13.4 (EMIC). Femurs were tested by the three-point bending flexural test, with force applied at a speed of 1.0 mm/min in the anterior-posterior direction. The gap between the two points was 8 mm, and a 2 Newtons (N) preload was used for 30 s.

### Histomorphometric Analysis

The femur was fixed in 10% formaldehyde, decalcified in 14% EDTA, and embedded in paraffin. Sections of 5 μm were stained with tartrate−resistant acid phosphatase (TRAP; Sigma−Aldrich, St. Louis, MO, United States). Osteoclast number over bone surface area (N.Oc/BS) and density of osteocytes per trabecular bone area were determined in the distal femur by using Adobe Photoshop C6 software (Adobe Systems, Inc., San José, CA, United States) and ImageJ software (NIH Image, Bethesda, MD, United States). Osteocyte and lacunae numbers were determined in a selected trabecular bone that was also quantified by measuring the total bone area in the analysis region. Osteocyte density was calculated by dividing the number of counted cells/lacunae by the area of trabecular bone.

Sections of the femur were also stained with Masson’s Trichrome. Osteoblast number per bone perimeter (N.Oc/B.Pm) was determined in the distal femur by using ImageJ software (NIH Image, Bethesda, MD, United States). Osteoblast number and the trabecular bone perimeter were quantified in the analysis region. Osteoblast density was calculated by dividing the number of counted cells (#)/the perimeter of trabecular bone (mm).

### Osteoclast Differentiation

Bone marrow cells (BMCs) were obtained from the cut shafts femur of mice. The remaining femur was placed in a perforated 0.5 mL microtube inserted in a 1.5 mL microtube, followed by centrifugation for 30 s at 10,000 rpm. The femur was discarded, and cells were washed with 1.0 mL of lysis buffer for 1 min and centrifuged for 5 min at 360 × *g*. The pellet was re-suspended in a 5.0 mL complete α-MEM medium. Cells were filtered using a cell strainer grid and counted on a Neubauer chamber. For osteoclast differentiation, BMCs were incubated in α-MEM medium (with no nucleosides; Thermo Fisher Scientific, Waltham, MA, United States) supplemented with 10% heat-inactivated fetal bovine serum (GIBCO, Carlsbad, CA, United States), 40 U/mL penicillin, and 40 mg/mL gentamicin. BMCs were seeded at 1 × 10^5^ cells/well in a 96 well plate. The medium was supplemented with soluble macrophage colony-stimulating factor (30 ng/ml; R&D Systems, Minneapolis, MN, United States) for 1 day, and RANKL (10 ng/ml; R&D Systems) for more 7 days. Cells were then fixed with formaldehyde 10% and stained with TRAP (Sigma-Aldrich) following the manufacturer’s instructions. TRAP-positive cells (with three nuclei or more) were counted, and results were expressed as the number of TRAP-positive cells per well.

### Serum Analyses

Glucose, triglyceride, and total cholesterol serum levels were measured by enzymatic kits (Bioclin, Belo Horizonte, Brazil). ELISA assay measured serum levels of Leptin, RANKL and OPG (R&D Systems Europe Ltd., Abington, United Kingdom) according to manufacturer instructions.

### mRNA Extraction and qPCR in the Femur

RNA extraction of the femur was performed using the Aurum total RNA mini kit (Bio-Rad, Hercules, CA, United States) and the samples were treated with DNAse as suggested by the manufacturer. The purity of RNA was checked spectrophotometrically, and the reverse transcription was carried out using the iScript cDNA synthesis kit (Bio-Rad, Hercules, CA, United States). The Real-time PCR assay was performed using the SYBR Green PCR Master Mix (Applied Biosystems) on an ABI PRISM 7500 sequence-detection system (Applied Biosystems, Warrington, United Kingdom). The relative gene expression of *Rankl* (*Tnfsf11*), *Opg* (*Tnfrsf11b*), and sclerostin (*Sost*) were assessed, determined by the 2-(ΔΔCt) method, and normalized by the housekeeping glyceraldehyde 3-phosphate dehydrogenase (*Gapdh*) expression. The primer sequences are listed in Supplementary Table S1.

### Osteocyte Cultures

MLO-Y4 osteocytes were cultured in α-minimal essential medium (α-MEM, GIBCO, Paisley, United Kingdom) supplemented with 10 μg/ml penicillin (Sigma-Aldrich, St. Louis, MO, United States), 10 μg/ml streptomycin (Sigma-Aldrich), 5% fetal bovine serum and 5% calf serum at 37°C in a humidified atmosphere of 5% CO_2_ in the air. The medium was replaced every 3 days, and upon reaching confluence, cells were harvested using 0.25% trypsin and 0.1% EDTA in PBS. The MLO-Y4 osteocytes were kindly provided by Dr. A. D. Bakker (Department of Oral Cell Biology, ACTA, Amsterdam, Netherlands). MLO-Y4 cells were seeded in 24- and 96-well plates for gene expression and proliferation/apoptosis assays, respectively.

#### Proliferation Assay

Proliferation rates were determined using the XTT method, according to the manufacturer’s instructions (Sigma-Aldrich). In short, MLO-Y4 osteocytes were plated in 96-well plates in α-MEM, at 30,000 cells per well in 100 μl. Cells were stimulated with 10–100 μg/ml milk EVs and cultured for up to 3 days at 37°C. After that, 50 μl XTT reagent was added to the wells, and plates were incubated at 37°C for 6 h. Spectrophotometrical absorbance was measured using a microplate reader (iMARK, Bio-Rad) at 490 and 650 nm as reference. Results were expressed as a fold-increase over time-matched controls.

#### Apoptosis Assay

Cell viability was determined using the above described XTT method, with a few modifications. MLO-Y4 cells were seeded in 96-well plates and stimulated with 10–100 μg/ml mEVs. After 24 h of stimulation, dexamethasone (10 μM) was added to induce cell apoptosis. After an additional 6 h, 50 μl XTT reagent was added to the wells, and plates were further incubated for up to 30 h in total. Spectrophotometrical absorbance was measured using a microplate reader at 490 and 650 nm as reference. Results were expressed as a percentage of PBS-treated controls (no dexamethasone).

#### Gene Expression

MLO-Y4 osteocytes were plated in 24-well plates in α-MEM, at 100,000 cells per well in 1 ml. Cells were stimulated with 100 μg/ml milk EVs and cultured for up to 48 h at 37°C. Total RNA was isolated from cells using TRI-reagent (Sigma-Aldrich) as described in the manufacturer’s protocol. The reference gene *Gapdh* was used to calculate the relative gene expression of fibroblast growth factor 2 (*Fgf2*), sclerostin (*Sost*), B-cell lymphoma 2 (*Bcl2*), Bcl2 associated × protein (*Bax*), *Opg*, and *Rankl* (Supplementary Table S1).

### Statistical Analysis

Results are expressed as means ± standard error of the mean (SEM) and analyzed using GraphPad Prism version 7.0 (GraphPad Software, San Diego, CA, United States). All data were analyzed for normality of distribution using the Kolmogorov–Smirnov test and were found to be normal. The one-way ANOVA followed by Dunnett and Newman–Keuls *post hoc* tests were used for obesity and ovariectomy models, respectively. A comparison between two groups was performed using the Student *t*-test. Two-way ANOVA followed by Bonferroni *post hoc* test was used in OGTT and Tb.Th distribution data. Statistical significance was set at *P* < 0.05.

## Results

### MEVs Reduce Bone Loss in the Diet-Induced Obesity Model

**FIGURE 1:**
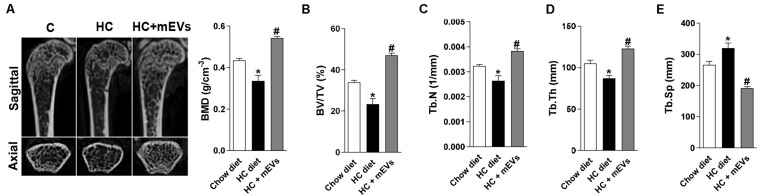
Micro-computed tomography analysis of trabecular bone in the femur of mice fed with high refined carbohydrate-containing (HC) diet and treated with milk extracellular vesicles (mEVs). **(A)** Representative pictures of distal femur microarchitecture and bone mineral density (BMD), **(B)** trabecular bone volume fraction (BV/TV), **(C)** trabecular number (Tb.N), **(D)** trabecular thickness (Tb.Th), **(E)** trabecular separation (Tb.Sp). Analyses were performed in mice that received (i) a standard diet (Chow diet), (ii) a HC diet, or (iii) HC diet treated with mEVs (HC + mEVs). Values are means ± SEM of 6–7 animals per group. * vs. Chow diet and # vs. HC diet group, *P* < 0.05.

**FIGURE 2:**
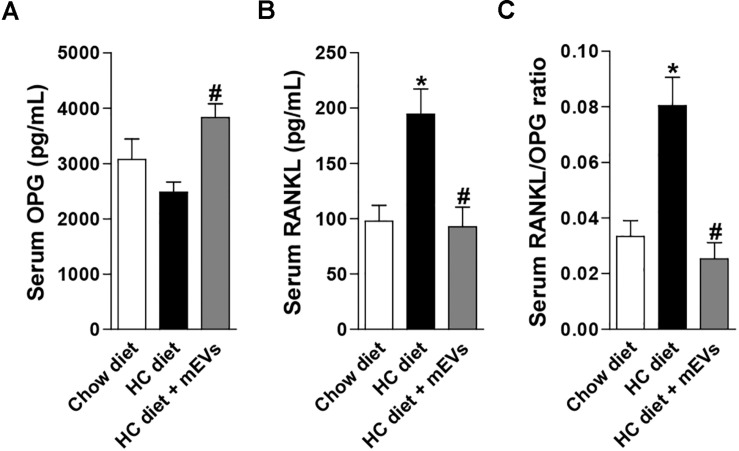
Evaluation of the RANKL/OPG system in the serum of mice fed with high refined carbohydrate-containing (HC) diet and treated with milk extracellular vesicles (mEVs). Serum levels of **(A)** osteoprotegerin (OPG), **(B)** receptor activator of nuclear factor κB ligand (RANKL), and **(C)** RANKL/OPG ratio. Analyses were performed in mice that received (i) a standard diet (Chow diet), (ii) a HC diet, or (iii) HC diet treated with mEVs (HC + mEVs). Values are means ± SEM of 6–7 animals per group. * vs. Chow diet and # vs. HC diet group, *P* < 0.05.

The mice fed with the HC diet showed bone loss features in the femur. A decrease in BMD (Figure 1A), BV/TV (Figure 1B), Tb.N (Figure 1C), Tb.Th (Figure 1D), and an increase in Tb.Sp (Figure 1E) was observed in the femur of mice fed with the HC diet compared with control. Interestingly, all parameters that changed in mice fed with the HC diet were reversed by mEV treatment (Figure 1). This model of obesity is characterized by no alteration in body weight gain (Supplementary Figure S1A), but an increase in visceral and subcutaneous adipose tissue (Supplementary Figure S1B) associated with increased leptin (Supplementary Figure S1C) in mice fed with HC diet. This higher fat mass also leads to metabolic alterations as glucose intolerance (Supplementary Figure S1D), hyperglycemia (Supplementary Figure S1E), and dyslipidemia (Supplementary Figures S1F,G). The treatment with mEVs did not alter body weight gain, adipose tissue mass, glucose intolerance, and total cholesterol levels compared with the HC diet group. Only glucose and triglycerides serum levels were reduced by mEV treatment (Supplementary Figure S1). Complementary to these metabolic alterations, although mice fed with HC showed no change in OPG serum levels (Figure 2A), they presented higher levels of serum RANKL (Figure 2B) and thereby an increased RANKL/OPG ratio (Figure 2C). The mEV treatment in HC diet-feeding mice contributed to an increase in OPG (Figure 2A) and reduction of RANKL serum levels (Figure 2B), leading consequently to a reduced RANKL/OPG ratio (Figure 2C) in this group.

### MEVs Increase the Femur Stiffness and Slightly Improves Femur Microarchitecture Parameters

**FIGURE 3:**
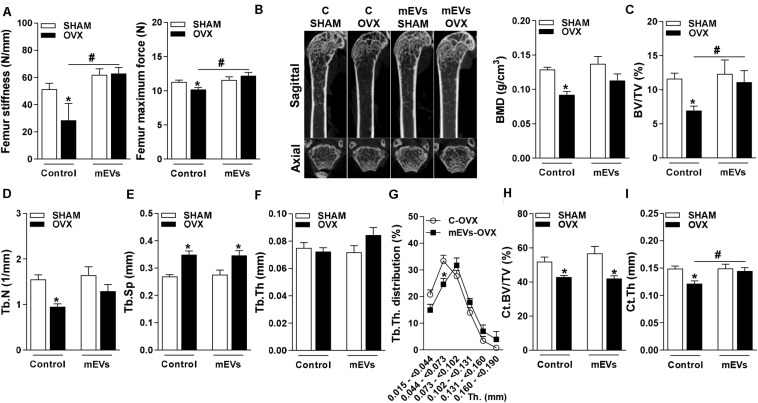
Mechanical analysis in the femur and micro-computed tomography analysis of trabecular and cortical bone in the distal femur of mice treated with milk extracellular vesicles (mEVs). **(A)** Stiffness and the maximum bone load of the femur. **(B)** Representative pictures of distal femur microarchitecture and bone mineral density (BMD), **(C)** trabecular bone volume fraction (BV/TV), **(D)** trabecular number (Tb.N), **(E)** trabecular separation (Tb.Sp), **(F)** trabecular thickness (Tb.Th), **(G)** trabecular thickness distribution (Tb.Th distribution), **(H)** cortical bone volume per tissue volume (Ct.BV/TV), and **(I)** cortical thickness (Ct.Th). Analyses were performed in mice (i) pseudo-operated (SHAM) or (ii) ovariectomized (OVX), (iii) non-treated (control), or (iv) treated with mEVs. Values are means ± SEM of 6–8 animals per group. * vs. respective SHAM group and # vs. non-treated group, *P* < 0.05.

To further study the potency of mEVs to reverse bone loss, as observed in the mild-obesity model, a more severe model of bone loss was performed using ovariectomized mice. The success of ovariectomy was determined by uterine atrophy. Ovariectomized (OVX) mice independent of treatment showed a similar reduction in the weight of ovary compared to pseudo-operated mice (SHAM) (C-SHAM 68.1 ± 4.5 mg vs. C-OVX 19.9 ± 1.6 mg, *P* < 0.05) and (mEVs-SHAM 62.8 ± 6.5 vs. mEVs-OVX 21.4 ± 2.6, *P* < 0.05). A three-point bending flexural test was performed to verify whether mEV treatment could alter the mechanical resistance of femurs from ovariectomized mice. The femoral stiffness and maximum force to fracture (Figure 3A) were reduced in OVX mice. Interestingly, treatment with mEVs prevents the loss of mechanical resistance shown in OVX non-treated mice (Figure 3A).

Several bone parameters were investigated by microCT to evaluate whether mEV treatment affects bone microarchitecture. Ovariectomized mice showed osteopenic effects on femur represented in the pictures of bone microarchitecture (Figure 3B), and as demonstrated by a decrease in multiple bone parameters: BMD (Figure 3B), BV/TV (Figure 3C), Tb.N. (Figure 3D), and an increase of Tb.Sp (Figure 3E). There was no difference in Tb.Th parameter between the groups (Figure 3F). However, Tb.Th stratification shifted by reducing significantly the amount of trabecular bone ranging from 0.044 to 0.073 mm in OVX mice treated with mEVs and incrementing higher ranges compared with non-treated mice (Figure 3G). The treatment with mEVs in OVX mice also induced an improvement BV/TV in femur microarchitecture compared C-OVX group (Figure 3C). The cortical BV/TV was reduced in both groups ovariectomized (Figure 3H). However, the reduction of Ct.Th observed in OVX mice was not found in OVX mice treated with mEVs (Figure 3I).

### The Use of mEVs Reduces the Number of Osteoclasts in the Femur and the Osteoclastogenic Potential of Precursors Cells in Ovariectomized Mice

**FIGURE 4:**
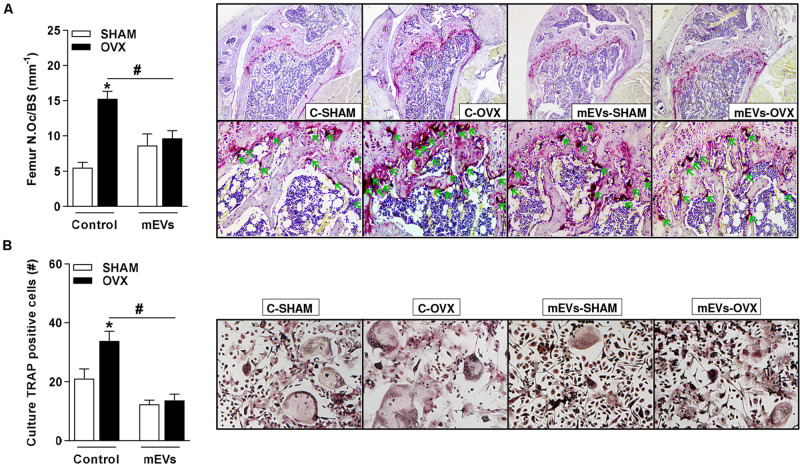
Effect of milk extracellular vesicle treatment on histological analyses of femur and osteoclast differentiation *in vitro*. **(A)** Osteoclast number over the bone surface area (N.Oc/BS) and representative histology of distal femur stained with TRAP (40× and 200×). Green arrows indicate osteoclasts. **(B)** The number of TRAP–positive cells/well and representative images of TRAP–positive cells in the culture (100×). Analyses were performed in mice (i) pseudo-operated (SHAM) or (ii) ovariectomized (OVX), (iii) non-treated (control), or (iv) treated with milk extracellular vesicles (mEVs). Values are means ± SEM of 6–8 animals per group. * vs. respective SHAM group and # vs. non-treated group.

Histological analyses of osteoclast presence by TRAP staining showed an increase in the number of osteoclasts in the femur (Figure 4A) of the C-OVX group compared with the C-SHAM group. In contrast, ovariectomized mice treated with mEVs presented a lower number of osteoclasts in the tissue evaluated (Figure 4A).

As an effect on osteoclast cells was observed in ovariectomized mice treated with mEVs, bone marrow cells from the femur were collected from all groups and maintained in an osteoclast differentiation medium. Ovariectomized mice showed an increase in the number of osteoclast TRAP-positive cells compared with the C-SHAM group (Figure 4B). Following the previous data, osteoclast numbers were reduced in wells with cells from ovariectomized mice treated with mEVs (Figure 4B).

### Systemic and Local Reduction in the RANKL/OPG Ratio by mEVs in Ovariectomized Mice May Be Regulated by Osteocytes

**FIGURE 5:**
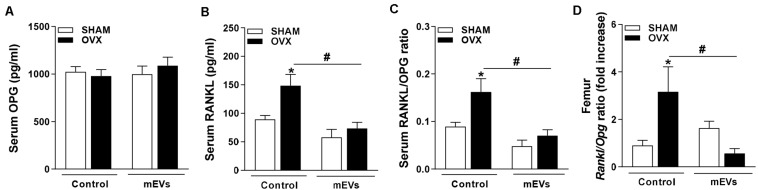
Effect of milk extracellular vesicle treatment in the OPG/RANKL axis in serum and femur. Serum levels of **(A)** osteoprotegerin (OPG), **(B)** receptor activator of nuclear factor κB ligand (RANKL), and **(C)** RANKL/OPG ratio. **(D)** mRNA expression of *Rankl*/*Opg* ratio in the femur. Analyses were performed in mice (i) pseudo-operated (SHAM) or (ii) ovariectomized (OVX), (iii) non-treated (control), or (iv) treated with milk extracellular vesicles (mEVs). Values are means ± SEM of 6–8 animals per group. * vs. respective SHAM group and # vs. non-treated group.

To understand the mechanisms involved in the effects of mEV treatment on osteoclast cells, the analysis of molecules involved in bone remodeling was performed. No significant differences in serum levels of OPG (Figure 5A) were found, but RANKL (Figure 5B) and RANKL/OPG ratio (Figure 5C) were higher in C-OVX group compared with C-SHAM group. Interestingly, ovariectomized mice treated with mEVs showed a decrease in both RANKL and RANKL/OPG ratio (Figures 5B,C). Similar to the systemic data, *Rankl*/*Opg* ratio expression in the femur was increased in OVX mice, and the treatment with mEVs reverted the ratio level (Figure 5D).

**FIGURE 6:**
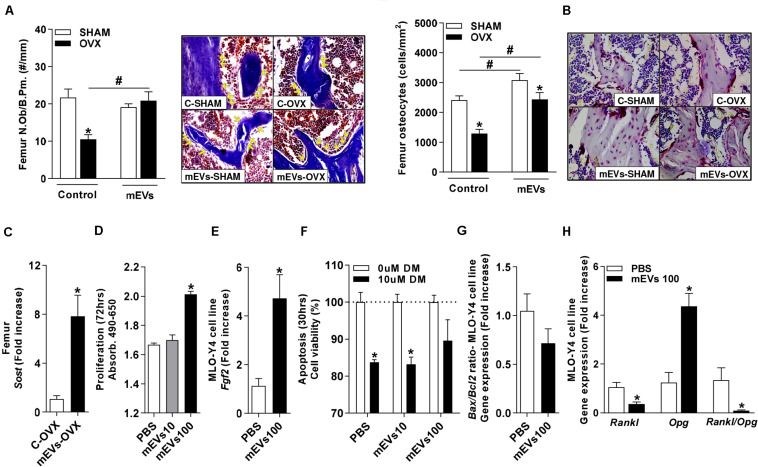
Effect of milk extracellular vesicle treatment in the osteocytes. **(A)** Number of osteoblasts (N.Ob) per bone perimeter (B.Pm) and representative pictures of osteoblasts in the trabecular bone (400×). Yellow arrows indicate osteoblasts. **(B)** Osteocyte density and representative pictures of osteocytes in the trabecular bone (400×). **(C)** mRNA expression of sclerostin in the femur. Analyses were performed in mice (i) pseudo-operated (SHAM) or (ii) ovariectomized (OVX), (iii) non-treated (control), or (iv) treated with milk extracellular vesicles (mEVs). Values are means ± SEM of 6–8 animals per group. * vs. respective SHAM group and # vs. non-treated group. *P* < 0.05. Osteocyte MLO-Y4 cell line **(D)** proliferation assay and **(E)** *Fgf2* mRNA expression. **(F)** Apoptosis assay of osteocyte cell line treated with dexamethasone (DM) (10 μM) and mEVs (10 and 100 μg/ml). mRNA expression of **(G)** *Bax*/*Bcl2* ratio and **(H)** *Rankl*, *Opg*, and *Rankl*/*Opg* ratio in osteocyte culture treated with mEVs (100 μg/ml) (*n* = 4). Bars represent the mean ± SEM. * vs. PBS. *P* < 0.05.

Osteocytes are the primary cells involved in the regulation of the RANKL/OPG system in bone tissues, and they are derived from osteoblasts. The osteoblast number in the trabecular bone of the femur was reduced in control mice OVX compared with the C-SHAM group. The treatment with mEVs in OVX mice increased the presence of these cells in the bone (Figure 6A). The osteocyte count in the femur (Figure 6B) revealed that the C-OVX group had fewer of these cells in the bone compared with the C-SHAM group. In the femur, the treatment with mEVs increased the number of osteocytes in both groups independent of ovariectomy, and it was, however, still reduced in OVX mice compared with the mEVs-SHAM group (Figure 6B). To confirm these data, the sclerostin expression, a marker for osteocytes, was evaluated in the bone. Its presence was increased in ovariectomized mice treated with mEVs compared with those not treated in the femur (Figure 6C).

To further evaluate whether mEVs could directly influence osteocytes, the osteocyte-like cell line MLO-Y4 was used (Kato et al., 2010). The increased osteocyte number observed *in vivo* could be due to either increased proliferation of osteocyte-precursor cells or mEVs beneficial effect on cell survival. To determine this, proliferation and apoptosis assays were performed, which revealed that a dose of 100 μg/ml mEVs improved proliferation (Figure 6D), associated with higher expression of *Fgf2* (Figure 6E) and *Sost* (PBS: 1.04 ± 0.08 vs. mEVs 100:41.2 ± 14.2 – fold increase, *P* > 0.05). Still, no alteration in cell viability response to dexamethasone treatment (Figure 6F) or expression of the *Bax*/*Bcl2* ratio (Figure 6G) was observed. Finally, the *Rankl* and *Opg* expression in response to mEV stimulation were determined. Similar to the *in vivo* model, osteocytes in culture also showed a significant decrease in *Rankl* expression and a substantial increase in *Opg* expression, resulting in a shifted *Rankl*/*Opg* ratio in mEVs-treated cells (Figure 6H).

## Discussion

Bone loss is associated with several diseases and may favor, at long-term, to a brittle bone, increasing the potential to fractures. Some conditions may contribute to the increased risk of this dysfunction, like aging, menopause (lack of estrogen), medicaments, low quality of food intake and unhealthy weight (Cao, 2011; Tarity et al., 2013; Cauley, 2015; Sakaguchi et al., 2019). Herein, we highlight two models of bone loss, mimicking obesity and menopause state, and evaluate the effect of mEVs as a treatment for them. The main results demonstrated that by using mEVs (i) the bone loss observed in mild-obesity induced by HC diet was reversed; (ii) osteoporotic mice showed a reduction of osteoclast presence in the femur, more stiffness, with improvement in microarchitecture parameters; (iii) in general both models were associated with an amelioration of systemic and local RANKL/OPG ratio; (iv) the primary cell regulator of RANKL/OPG system, osteocytes, were increased in the bone of osteoporotic mice; (v) the stimulus with mEVs in MLO-Y4 cell line led to a modulation of *Rankl* and *Opg* expression also reducing the *Rankl*/*Opg* ratio.

Obese and diabetic patients demonstrated reduced BMD independent of age, increasing the risk of fractures (Leslie et al., 2018; Samelson et al., 2018; Lee et al., 2019). Studies have demonstrated that the consumption of diets rich in sucrose and fat aggravates the bone loss or change negatively bone cell activity and mineral metabolism in animals (Dong et al., 2016; Lavet et al., 2016). As shown before, mice fed with the HC diet have adipose tissue expansion and metabolic alterations associated with systemic and local inflammation (Oliveira et al., 2013). Following the present study, this model was previously associated with bone loss in the femur when mice were fed with the HC diet for 12 weeks (Montalvany-Antonucci et al., 2018). Studies have investigated different ways to prevent the progression of bone loss as pharmacological interventions (Delmas et al., 2003; Engelke et al., 2015) or dietary supplementation (Du et al., 2004; Harkness et al., 2004). Nutritional components are influential in the development and treatment of osteoporosis, especially calcium and vitamin D intake (Feskanich et al., 2003). The present work proposed an intervention through the oral administration of mEVs, and our data demonstrated that they have the potential to protect bone loss in diet-induced obesity and ovariectomized mice. These nanoparticles have already shown the ability to alter the response of bone cells, especially *in vitro*, with the modulation of their process of differentiation and activity (Oliveira et al., 2016, 2017). The effect of milk on bone has been extensively studied, and still, a controversial association regarding consumption of it or its components and maintenance of bone tissue is observed (Kalkwarf et al., 2003; Uenishi et al., 2007; Bonjour et al., 2008; Michaëlsson et al., 2014). We believe that milk has a complex composition and this could contribute to its undefined function on bone as well as the numerous experimental design used. Thus, the isolation of a component might increase its possibilities of application.

The bone marrow is a heterogeneous regulatory niche consisting of multipotent progenitors, including mesenchymal stromal cells (MSC). The MSC interact with, and are regulated by osteoblast lineage cells such as osteocytes, and may undergo a shift in their cellular line throughout their engagement to terminal differentiation (Pierce et al., 2019). We observed a reduction in osteoblast and osteocyte number in OVX mice after the treatment with mEVs, which was increased in non-treated mice. MEVs were already shown to have the potential to increase osteoblast differentiation of human mesenchymal stem cells into osteocytes (Oliveira et al., 2016). These data indicate that mEVs are prone to stimulate osteoblast linage and may provide benefits in bone loss disorders. MSCs orchestrate bone homeostasis by regulating both bone-forming osteoblasts (Nakashima et al., 2011), but they may also alternatively be directed to adipogenesis under certain conditions. Studies have demonstrated that stem cells niche is affected by obesity-induced chronic inflammation, and may trigger a competition between these differentiation pathways, promoting an increase in adipogenic precursors (Ye and Gimble, 2011). In our study, the induced bone loss in mice receiving diet-induced obesity was reversed by the treatment with mEVs, even with a not so strong impact on obesity features. Although the HC diet might increase the inflammation in the stem cells niche, we consider that mEV treatment could affect it, resulting in an enhancement of MSC turnover into the osteogenic lineage. However, further research is required to provide evidence of whether mEVs affect the stemness and the osteoblast/osteocyte differentiation process through the evaluation of the stemness of MSCs or the inflammatory response of obesity.

Osteoclasts are cells derived from hematopoietic tissue and are responsible for bone resorption during the remodeling process of this tissue (Wada et al., 2006). The increased number of TRAP-positive cells in the femur and higher osteoclast differentiation in cultured cells isolated from bone marrow in OVX mice was reduced in the group treated with mEVs. RANKL specifically regulates osteoclastogenesis by triggering osteoclast formation, and OPG is counter-regulatory in this process (Wada et al., 2006). Our findings indicate that mEVs positively act on the RANKL/OPG system by decreasing their ratio systemically and locally. These data corroborate with other studies, in which a decrease in this ratio is associated with reduced osteoclastogenesis after treatment with plant and root extracts in models of bone loss, such as ovariectomy (Sun et al., 2019; Zhu et al., 2020) or glucocorticoid (Mo et al., 2019). An amelioration in bone structure with the treatment of mEVs was shown by improving the bone stiffness despite some slight effect in the femur microarchitecture in OVX mice. The major limitation of this model is the intervention duration. Previous studies have shown that OVX animals treated with herbal medicine for 12 weeks (Li et al., 2016) or with *Labisia pumila* extract for 3, 6, and 9 weeks (Effendy et al., 2014) did not completely reverse bone loss, but a considerable improvement was observed. It is suggested that a longer treatment period is necessary to induce greater phenotypic changes despite the molecular alterations present (Effendy et al., 2014). Then, we believe that an experiment with a more extended intervention period is required to demonstrate an effect of mEVs on osteoporosis that enables a complete delay of bone loss microarchitecture. However, the lower amount of osteoclasts observed in the mEV treated group may be an indicator of a long-term improvement in bone loss.

As previously shown, mEVs accelerate the differentiation of mesenchymal stem cells into osteocytes, associated with more proliferation of cells *in vitro* (Oliveira et al., 2016). As we also observed more proliferation of osteocytes *in vitro* in this study, the increase in osteoblast differentiation and proliferation might be one of the factors that may lead to augmented osteocyte number in the bone of mEVs-OVX group. Osteocytes are considered the last stage in the differentiation of osteoblasts and act as sensory cells for transporting information between osteoclasts and osteoblasts, contributing to the maintenance of the bone matrix (Palumbo et al., 2001). These cells also produce RANKL and OPG, critical regulators of osteoclastogenesis, as aforementioned (Nakashima et al., 2011; Xiong et al., 2011). We observed a reduction in osteoblast and osteocyte presence in the femur of ovariectomized mice that was reversed by mEV treatment. An increase of osteocyte apoptosis after ovariectomy (Emerton et al., 2010) caused by the lack of estrogen (Huber et al., 2007; Mann et al., 2007) or glucocorticoid treatment (Weinstein et al., 1998; Plotkin et al., 2007) was already described, and its reversion contributes to reduced bone resorption (Emerton et al., 2010). The bone loss was suggested to be due to a higher release of RANKL from apoptotic osteocytes or induction in neighboring cells, although not so well established how it works (Kennedy et al., 2012). The importance of osteocytes was also highlighted by using bisphosphonates that prevent osteocyte apoptosis and protect the loss of bone strength induced by glucocorticoid (Plotkin et al., 2011). Although we could not observe a direct effect of mEVs in osteocyte apoptosis *in vitro*, ovariectomized-mice treated by mEVs demonstrated higher bone stiffness compared to those not treated, confirming the importance of these cells in bone maintenance. Our data indicate that the treatment with mEVs might have a substantial effect on cell turnover by increasing the proliferation of cells rather than reducing apoptosis. It may be that the vesicles contribute to the RANKL/OPG balance and provide the osteocyte homeostasis, maintaining the regulation of bone resorption and bone strength.

Herein, we demonstrated that mice fed with HC diet, a model of mild-obesity with bone loss, and ovariectomized mice, a model of osteoporosis, showed protection of bone loss when the treatment with mEVs occurred. This improvement appears to be related to the positive regulation of the RANKL/OPG system by osteocytes. Therefore, these data demonstrate the potential of mEVs to influence cells related to bone remodeling and indicate an innovative therapeutic product against bone loss, creating the possibility to progress, particularly in the area of food-illness interaction.

## Data Availability Statement

The raw data supporting the conclusions of this article will be made available by the authors, without undue reservation.

## Ethics Statement

The animal study was reviewed and approved by the Comissão de Ética no Uso de Animais (CEUA) – Universidade Federal de Minas Gerais (UFMG).

## Author Contributions

MO conceived and designed this study. MO, BP, PG, LD, JH, AS, AO, and SM performed the experiments and analyzed the data. MO, BP, MT, AF, TS, FL, and SM provided the resources and wrote the manuscript. All authors read and approved the manuscript.

## Conflict of Interest

The authors declare that the research was conducted in the absence of any commercial or financial relationships that could be construed as a potential conflict of interest.
