# Supplementary material for: Bovine Milk Extracellular Vesicles Are Osteoprotective by Increasing Osteocyte Numbers and Targeting RANKL/OPG System in Experimental Models of Bone Loss
Source: Front Bioeng Biotechnol. 2020 Jul 31;8:891. doi: 10.3389/fbioe.2020.00891 (PMC7411003; doi:10.3389/fbioe.2020.00891)
Supplement: Supplementary file 1 [file Image_1.pdf]

Supplementary Material

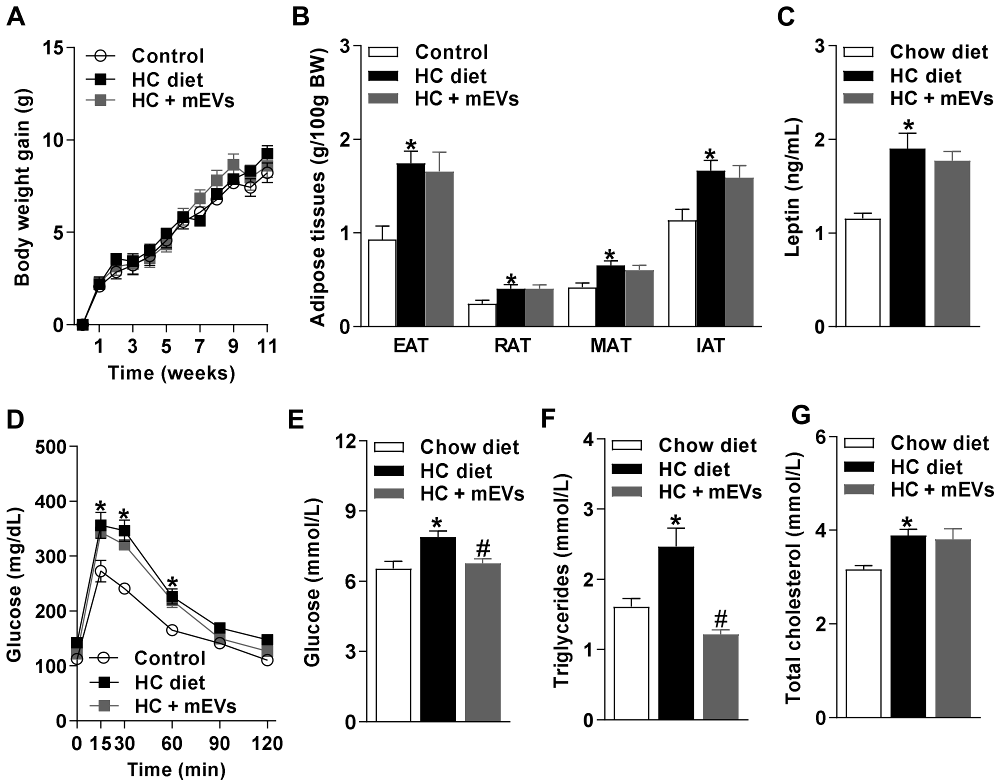

Supplementary Figure S1. Obesity features induced by a high refined carbohydrate-containing (HC) diet and the effect of milk extracellular vesicles (mEVs) on them. (A) Body weight gain, (B) epididymal (EAT), retroperitoneal (RAT), mesenteric (MAT) and inguinal (IAT) adipose tissues, (C) serum leptin levels, (D) oral glucose tolerance test. Serum levels of (E) glucose, (F) triglycerides and (G) total cholesterol. Analyzes were performed in mice that received (i) a standard diet (Chow diet), (ii) a high refined carbohydrate-containing (HC) diet, or (iii) HC diet treated with milk extracellular vesicles (HC + mEVs). Values are means ± SEM of 6-7 animals per group. *vs. Chow diet and #vs. HC diet group. $P < 0.05$.
