# Supplementary material for: Bovine Milk Extracellular Vesicles Are Osteoprotective by Increasing Osteocyte Numbers and Targeting RANKL/OPG System in Experimental Models of Bone Loss
Source: Front Bioeng Biotechnol. 2020 Jul 31;8:891. doi: 10.3389/fbioe.2020.00891 (PMC7411003; doi:10.3389/fbioe.2020.00891)
Supplement: Supplementary file 2 [file Table_1.docx]

Supplementary Material

# Supplementary Table S1 - Primers used for quantitative PCR in the femur and osteocyte cells.

| Gene | Forward | Reverse |
| --- | --- | --- |
| Gapdh (femur) | 5’-ACGGCCGCATCTTCTTGTGCA-3’ | 5’-CGCCAAATCCGTTCACACCGA-3’ |
| Rankl (femur) | 5’-CGTGCAGAAGGAACTGCAACAC-3’ | 5’-TGGTGAGGTGTGCAAATGGCT-3’ |
| Opg (femur) | 5'-TCATCCAAGACATTGACCTCTGTGA-3' | 5'-GCTGCTCGCTCGATTTGCAG-3' |
| Sost (femur) | 5'- TGTCAGGAAGCGGGTGTAGT -3' | 5'- GAGCCTCCTCCTGAGAACAA -3' |
| Gapdh (cells) | 5’-GGCAAATTCAACGGCACA-3’ | 5’-GTTAGTGGGGTCTCGCTCCTG-3’ |
| Fgf2 (cells) | 5’-GGCTGCTGGCTTCTAAGTGT-3’ | 5’-TCTGTCCAGGTCCCGTTTTG-3’ |
| Sost (cells) | 5’-GGAATGATGCCACAGAGGTCAT-3’ | 5’-CCCGGTTCATGGTCTGGTT-3’ |
| Bcl2 (cells) | 5’-GCATGCGACCTCTGTTTGAT-3’ | 5’-ATTTGTTTGGGGCAGGTTTGT-3’ |
| Bax (cells) | 5’-TGAAGACAGGGGCCTTTTTG-3’ | 5’-AATTCGCCGGAGACACTCG-3’ |
| Rankl (cells) | 5’-ctgaggcccagccatttg-3’ | 5’-gttgcttaacgtcatgttagagatcttg-3’ |
| Opg (cells) | 5’-TGCTCCTGGACATCATTG-3’ | 5’-CCTTCCTCACACTCACACAC-3’ |
